# Functionalization of Cotton Fabrics with Polycaprolactone Nanoparticles for Transdermal Release of Melatonin

**DOI:** 10.3390/jfb9010001

**Published:** 2017-12-24

**Authors:** Daniele Massella, Federica Leone, Roberta Peila, Antonello A. Barresi, Ada Ferri

**Affiliations:** 1 Department of Applied Science and Technology, Politecnico di Torino, Corso duca degli Abruzzi 24, 10129 Turin, Italy; daniele.massella@polito.it (D.M.); federica.leone@polito.it (F.L.); roberta.peila@polito.it (R.P.); antonello.barresi@polito.it (A.A.B.); 2 University Lille Nord de France, F-5900 Lille, France; 3 ENSAIT, GEMTEX, F-59100 Roubaix, France; 4 College of Textile and Clothing Engineering, Soochow University, Suzhou 215123, Jiangsu, China

**Keywords:** textile, cotton, melatonin, nanoparticles, drug delivery, Franz cell, PCL, DSC, flash nanoprecipitation (FNP), pharmaceutical nanotechnology

## Abstract

Drug delivery by means of transdermal patches raised great interest as a non-invasive and sustained therapy. The present research aimed to design a patch for transdermal delivery of melatonin, which was encapsulated in polycaprolactone (PCL) nanoparticles (NPs) by employing flash nanoprecipitation (FNP) technique. Melatonin-loaded PCL nanoparticles were successfully prepared with precise control of the particle size by effectively tuning process parameters. The effect of process parameters on the particle size was assessed by dynamic light scattering for producing particles with suitable size for transdermal applications. Quantification of encapsulated melatonin was performed by mean of UV spectrophotometry, obtaining the estimation of encapsulation efficiency (EE%) and loading capacity (LC%). An EE% higher than 80% was obtained. Differential scanning calorimetry (DSC) analysis of NPs was performed to confirm effective encapsulation in the solid phase. Cotton fabrics, functionalized by imbibition with the nano-suspension, were analyzed by scanning electron microscopy to check morphology, adhesion and distribution of the NPs on the surface; melatonin transdermal release from the functionalized fabric was performed via Franz’s cells by using a synthetic membrane. NPs were uniformly distributed on cotton fibres, as confirmed by SEM observations; the release test showed a continuous and controlled release whose kinetics were satisfactorily described by Baker–Lonsdale model.

## 1. Introduction

Skin is the largest tissue of the human body (about two square meters) and accomplishes different important roles, such as body protection from external factors (i.e., pathogenic agents) [1]. Furthermore, skin has been used for several decades as route of administration for both local and systemic drugs. In accordance to Food and Drug Administration (FDA), the former case is defined topical administration and consists in the application of a drug to a particular spot on the outer surface of the body; the latter is defined transdermal administration and involves the delivery through the dermal layer of the skin to the systemic circulation by diffusion. Notwithstanding the numerous advantages in using skin for topical and transdermal drug delivery, skin administration of several compounds is still a challenging issue, due to the structural complexity of the skin barrier [2]. Nowadays, nanotechnology provides a promising solution to overcome this problem [3]. Biofunctional textiles [4]—a new class of materials based on the combination of conventional textiles with advanced drug delivery systems [5]—have brought about significant improvements in the transdermal administration of active molecules [6,7]. Among biofunctional textiles, innovative transdermal patches have gained considerable attention, due to the possibility to improve the performance of these devices by their functionalization with nanocarrier-based drug delivery systems [5,8]. Transdermal patches aim at improving drug permeation through the skin barrier, reaching blood circulation. This approach ensures a sustained and constant drug release, with non-invasive delivery, reducing drug toxicity.

Biofunctional textiles composed by natural fabrics have been attracting much interest because of their improved compatibility, which opens new utilizations in the biomedical field. Moreover, natural fibers are largely available, biodegradable, and represent renewable resources in comparison with synthetic fibers. For all the above mentioned advantages, cotton is a natural cellulosic fiber widely used in the preparation of biofunctional textile [9,10]. However direct immobilization of an active compound onto the fabrics surface may cause some drawbacks such as scarce skin penetration or alteration of drug activity; for this reason many authors proposed to encapsulate the drug in a nanocarrier prior to the functionalization of the textile material [5,11].

Nanocarriers represent one of the most studied transdermal drug delivery system [12,13]. Polymeric nanoparticles are among the most studied nanocarriers for drug delivery application, since they have shown superior bioavailability and encapsulation yield; moreover, they can delivery effectively therapeutic dose while minimizing side effects [14]. The polymeric materials must fulfill specific requirements such as being non-toxic, biocompatible and biodegradable [15]. The list of polymers with such characteristics includes poly-lactide-*co*-glycolide, polylactic acid, poly-ε-caprolactone, chitosan and gelatin [16].

Poly-ε-caprolactone (PCL) is a synthetic polymer that, thanks to its intrinsic biocompatibility and biodegradability, is widely exploited in several biomedical applications such as tissue engineering, implantable devices and nanomedicine [17,18]. PCL is mainly biodegraded in body fluids by cleavage of the ester bond. The degradation mechanism consists in a first step in which PCL molecular weight decreases due to chain scission and a second one where an actual weight loss is observable; the overall biodegradation process can take up to one year to be completed and this makes PCL suitable for a wide range of applications requiring long lasting releases [19]. Due to its versatility as drug carrier, poly-ε-caprolactone has also been investigated for the transdermal route [20,21]. 

Concerning the production of PCL nanoparticles, a wide range of techniques are currently used at research level. These methods include solvent evaporation [22], double emulsion [23], emulsion polymerization [24], spray drying [25], and solvent displacement [26]. Among the above mentioned technologies, a particular version of the solvent-displacement approach called flash nanoprecipitation (FNP) [27] has garnered interest in terms of reproducibility and control, two key factors for scale-up to industrial production [28].

In nanoprecipitation the preformed polymer is solubilized in a suitable organic solvent, and the polymeric solution is then mixed with an anti-solvent in which the polymer is not soluble; the mixing of the two liquid results in polymer precipitation in nanoparticle form [29]. The mixing condition is a key factor in controlling nanoparticle formation; for this reason micro-mixers and micro-reactors are used, especially when fast phenomena are occurring [27]. Micro-mixers are systems with characteristic length scale from 1 to 1000 µm in which it is possible to achieve fast homogenization of the two streams and good control of process parameters; Confined Impinging Jets Mixers (CIJM), in particular, have been tested in several experimental works to produce polymeric nanoparticles [30,31,32,33,34]. Moreover, many modelling studies have been conducted in order to understand the dynamics of FNP in CIJM and this makes this technique sound from the point of view of process control [35,36,37]. 

It has been shown that FNP is an effective technique to encapsulate drugs of different type such has menthol [33], curcumin [38], paclitaxel [39] and vitamin E [40]; these active principles are characterized by a marked hydrophobic behavior (LogP > 3.2, where P, partition-coefficient is the ratio of concentrations of a compound in a mixture of two immiscible phases at equilibrium).

Generally speaking, the difficulties encountered in encapsulating hydrophilic substances are considered one of the weaknesses of FNP technique [41], while recent studies are tackling the issue of employing FNP to encapsulate more hydrophilic substances (LogP < 1.6) [42,43,44]. 

Among the numerous active pharmaceutical ingredients with a degree of hydrophilicity suitable for encapsulation by FNP, melatonin (LogP = 1.6) is of particular interest in the field of bio functional textiles [5]. Melatonin (MEL) is an hormone produced by the pineal gland, regulating the circadian rhythm in mammals [45]. Traditionally, melatonin is orally administered to enhance daytime sleep quality and reduce jet lag. Moreover, it is claimed to possess analgesic, anti-oxidative and anti-inflammatory effects [13]. Despite the conventional administration route of melatonin being the oral one, this involves several drawbacks affecting its efficiency. For instance, the oral fast-release, followed by scarce bioavailability due to first pass metabolism, affects the duration of efficient melatonin plasma levels decreasing the sleep-promoting effect. Moreover, the relatively high oral dose of melatonin can cause the receptors desensitization, leading to tolerance insurgence [46]. In order to overcome these limitations, alternative administration routes for melatonin are under investigation. Melatonin has been indicated as a good candidate for transdermal drug delivery in numerous investigations [47,48], proposing skin as successful alternative to the oral route.

The present research work aimed at the development of an innovative patch for transdermal delivery of melatonin. Given the necessity to enhance skin penetration and preserve bioactivity of melatonin, it was decided to encapsulate it in a polymeric nanoparticles system that could penetrate the skin outer layer and slowly release the drug in the inner one. For this reason it was decided to incorporate MEL in PCL nanoparticles by using a simple and scalable approach such as FNP. Firstly, melatonin-loaded nanoparticles were produced in CIJM, then a careful characterization of the drug-loaded system was performed, in order to determine its features, such as sizes and loading capacity, estimating the encapsulation efficiency of the process. Secondly, cotton fabrics were functionalized by imbibition and their morphology was observed via scanning electron microscopy. In conclusion, melatonin delivery from the functional fabric was assessed by Franz’s cell release test.

## 2. Materials and Methods

### 2.1. Materials

All chemicals were purchased from Sigma Aldrich (St. Louis, MO, USA). Melatonin was in powder form with assay ≥98%, PCL flakes with an average molecular weight of 14,000 Da were used. Acetone with purity ≥99.5% meeting European Pharmacopedia standards was employed as solvent. Phosphate buffer solution was prepared by using the following salts: sodium chloride anhydrous ≥99%, potassium chloride ACS grade ≥99.5%, sodium phosphate dibasic dehydrate ≥99% and potassium dihydrogen phosphate ACS reagent ≥99%. Ultrapure water was produced by mean of a Milli-Q RG system by Millipore R (Billerica, MA, USA) and used in all experiments. The skin mimicking membrane for the Franz diffusion cell was made of mixed cellulose esters with 0.45 μm pore size, purchased from Whatman knitted cotton fabrics (100% cotton, Nm 30/1, single jersey) were kindly gifted by Eusebio S.p.A (Crugnola di Mornago (VA), Italy). The fabrics were scoured at 95 °C for 30 min in a 4 mg/mL Na_2_CO_3_ water solution and rinsed under tap water for 10 min to remove impurities and waxes.

### 2.2. Nanoparticles Preparation

**Figure 1:**
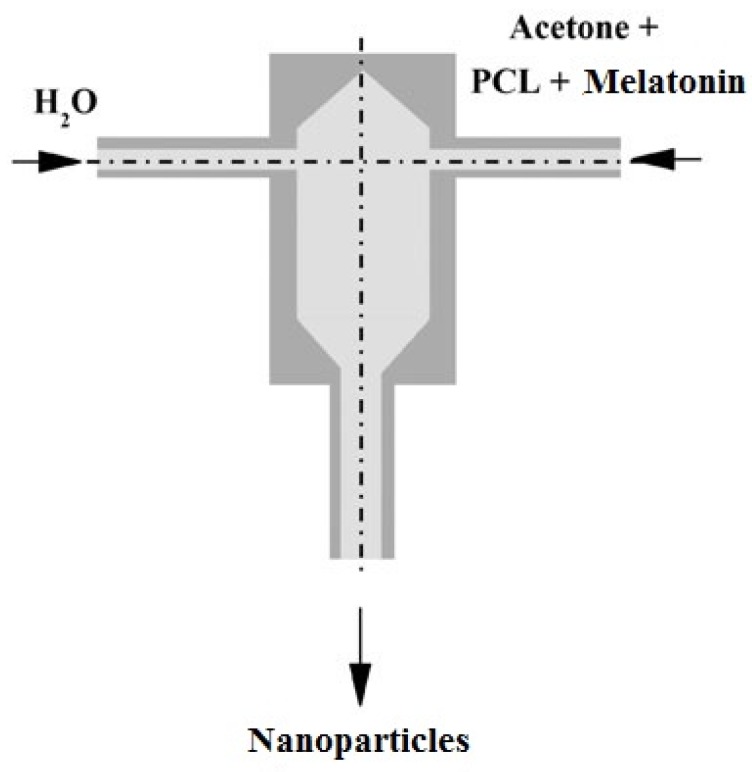
Scheme of the process of nanoparticles production by flash nanoprecipitation (FNP).

The nanoparticles were prepared by the flash nanoprecipitation technique in a confined impinging jets mixer with the following characteristics: 1 mm inlet tube diameter, 5 mm chamber diameter, 11.2 mm chamber height. The process is sketched in Figure 1: a stream of polymer in organic solvent solution is mixed with an anti-solvent, in this case water. The collision of the two jets induces polymer precipitation in the form of nanoparticles. 

PCL was dissolved in acetone at concentration from 6 to 25 mg/mL and melatonin was dissolved in the same solution at concentrations from 4.56 to 36 mg/mL. A complete summary of all formulations is reported in Table 1: in the last column MR, namely the drug-to-polymer mass ratio, is given. The proper volume of acetone solution and anti-solvent were placed in syringes and fed to the CIJM micro-mixer by a syringe pump (KDS200, KD Scientific, Holliston, MA, USA) at a flow rate from 5 to 120 mL/min. Sample of 8 mL (4 mL for each stream) were taken, collected in a glass vial containing 4 mL of quenching water placed downstream of the mixer; the collected suspension and the quenching water were kept under magnetic stirring for two minutes and then the nanoparticle suspension was sent to characterization and analysis.

### 2.3. Nanoparticle Characterization

#### 2.3.1. Dynamic Light Scattering

Size distribution and Zeta potential were measured by means of DLS Zetasizer Nanoseries ZS90, Malvern Instruments (Malvern, UK). Samples were prepared by diluting 0.1 mL of nanoparticles suspension in 1 mL of ultrapure water. All samples were measured in triplicate under controlled temperature at 25.0 ± 0.1 °C. 

#### 2.3.2. Determination of Loading Capacity (LC) and Encapsulation Efficiency (EE)

Loading capacity was defined as the mass of encapsulated (m_en_) drug divided by the mass of the whole polymeric nanoparticles system (m_tot_) as given by Equation (1); it is an indicator of the amount of drug that can be incorporated in a given amount of nanoparticle formulation. Encapsulation Efficiency was defined as the amount of encapsulated melatonin over the input quantity of melatonin in the process (m_in_) as expressed by Equation (2); it is an index of the efficiency of the nanoparticles production process.

LC = m_en_/m_tot_ (1)

EE = m_en_/m_in_ (2)

In order to calculate LC% and EE% the following method based on UV-spectroscopic analysis was developed. The suspension was placed in a rotary evaporator RE 300 at 50 °C, under vacuum for 15 min, to remove acetone; then, it was centrifuged for 10 min at 19,700 g in a SL 16 Thermo scientific centrifuge and the liquid phase was finally separated from the solid NPs. 

The 0.1 mL supernatant sample was diluted in 50 mL of water, filtered through a cellulose syringe filter of porosity of 0.2 µm and analyzed via UV-Vis spectroscopy. EE% and LC% were calculated by quantification of free melatonin in the supernatant by mean of a 6850 UV/Vis spectrophotometer, Jenwa (Stone, Staffordshire, UK). The solid nanoparticles were dried and sent to DSC analysis.

#### 2.3.3. Differential Scanning Calorimetry

Samples of PCL, MEL and MEL-loaded nanoparticles were analyzed by mean of a DSC Q 200 TA instruments (Milano, Italy). In the case of nanoparticles samples, care was taken to separate them from the liquid after centrifugation and drying before performing the analysis. The analysis was conducted in nitrogen atmosphere; samples were stabilized at 20 °C and heated to 150 °C with a ramp of 5 °C/min.

### 2.4. Fabric Functionalization

Cotton fabrics were functionalized by imbibition. A volume of 0.5 mL of freshly prepared nanoparticles suspension was employed for the functionalization of the fabric disk. Each cotton disk has a 2.5 cm diameter. The suspension was added dropwise, taking care that each drop was completely absorbed by the fabric and that the colloidal system was uniformly spread all over the surface of the cotton disk.

After the desired suspension volume was delivered to the fabric, the samples were dried in standard textile laboratory conditions (21 °C and 65% RH) for 8 h.

### 2.5. Scanning Electron Microscopy

The fabrics surface morphology was examined by a Leica Electron Optics 435 VP scanning electron microscope (Cambridge, UK) with an acceleration voltage of 15 kV, a current probe of 400 pA and a working distance of 20 mm. The samples were mounted on aluminum specimen stubs and sputter-coated with gold in rarefied argon atmosphere using an Emitech K550X Sputter Coater (Ashford, Kent, UK) with a 20 mA current for 240 s.

### 2.6. In Vitro Release Test

The in vitro release test from the functionalized fabrics was conducted on static vertical Franz diffusion cells (PermeGear, Hellertown, PA, USA).

The Franz cells were constituted by an upper donor chamber and a lower receptor chamber (volume 12 mL) with a contact surface of 1.8 cm^2^. The acceptor compartment was filled with pH 7.4 phosphate buffer solution kept at temperature of 33 °C by a heating jacket. 

The skin mimicking membrane was boiled for 60 min to remove the glycerol size and enhance its wettability; finally, the fabric patches were placed over the membranes in the donor compartment and sealed over the membrane with a clamp. Samples of the acceptor fluid were withdrawn at a fixed time interval and analyzed for melatonin by spectrophotometry; after withdrawal, an equal volume of the fresh PBS solution was replaced into the cell.

### 2.7. Release Kinetics Modeling

The melatonin release data were fitted by Zeroth-Order, First-Order, Higuchi, Hixon–Crowell, square root of mass, three seconds root of mass and Baker–Lonsdale models [49,50]. The regression was performed by interpolating the data of the curve until the asymptote in the cumulative release was reached.

The determination coefficient was used to discriminate the best fitting model.

The model equations are listed in Table 2 with *F* denoting the fraction of total drug released up to time *t* and *k* the kinetic constant of each model.

## 3. Results and Discussion

### 3.1. Nanoparticles Size

The particle size is a crucial factor in the design of a transdermal patch; in fact, the particle diameter differentiates whether the particle will penetrate the epidermis to deliver the drug inside the skin or whether it will stick to the fabric and elute the active substance from it. To achieve an effective control on the particle size, the role of key process parameters, namely inlet flow rate, PCL and melatonin concentration in the impinging stream, were studied.

Only batches presenting mono-modal distribution of NP size, which met instrumentation quality criteria, were further considered for fabric functionalization. 

#### 3.1.1. Effect of Flow Rate

DLS analysis showed that diameter decreased exponentially as the inlet flow rate increases up to a threshold value of about 80 mL/min, above which the NP size tended to oscillate around an asymptotic value. Such trend is in accordance with our previous data [29,33,51,52] for loaded and unloaded NPs; moreover, the same phenomenon has been extensively observed in the literature [27,30].

To minimize the diameter as required for transdermal applications, further experiments were conducted at 80 mL/min, namely the threshold value above which NP size was levelled off.

#### 3.1.2. Effect of Melatonin and Polymer Initial Concentration

**Figure 2:**
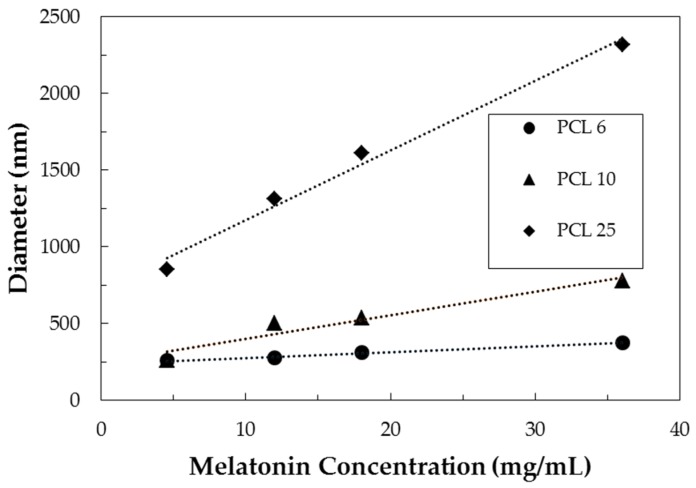
Particle size versus inlet PCL and melatonin concentration for samples produced at FR = 80 mL/min.

The NP diameter is plotted against initial melatonin concentration for different initial PCL concentrations in Figure 2. A linear increase of NP diameter was observed as initial melatonin concentration increased but this effect was more pronounced as PCL concentration increased; generally speaking, the particles produced with higher polymer concentration are larger.

These trends can be explained considering separately the contribution of the two species. The addition of melatonin to the formulation caused the particle diameter to increase as a consequence of the effective incorporation of the drug inside the polymeric system (as discussed in Section 3.3). On the other hand, increasing PCL concentration made more polymer available during the growth stage of the nanoparticle, resulting eventually in particles of micrometric size. 

### 3.2. Zeta Potential

**Figure 3:**
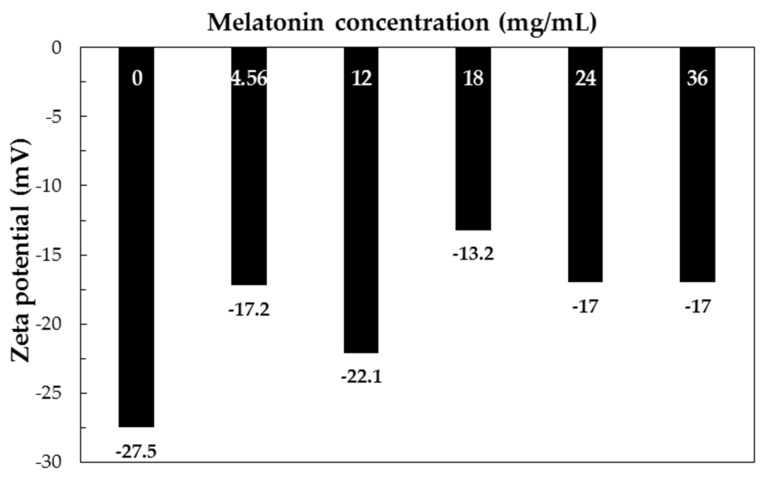
Zeta potential of formulations with PCL 6 mg/mL and different melatonin concentrations (FR 80 mL/min).

The zeta potential for different melatonin concentrations are shown in Figure 3.

A precise trend cannot be evidenced; however, a slightly more positive charge was observed in melatonin-loaded NPs with respect to plain PCL NPs. This effect can be explained by observing that melatonin possesses amine groups, which can undergo protonation in aqueous environment; thus, the presence of free (not encapsulated) melatonin in the colloidal system can induce the increase of zeta potential.

### 3.3. Loading Capacity and Encapsulation Efficiency

LC% and EE% were determined for some selected samples size as summarized Table 3.

High process yields were always achieved, leading to minimum depletion of active substance during nanoparticle production. Samples produced with low initial melatonin concentration presented the lowest EE%. This is not surprising and was ascribed to melatonin aqueous solubility (~2 mg/mL), which is not negligible. In fact, some melatonin is transferred and dissolved in water during the flash precipitation process and this amount is in percentage more relevant for low initial melatonin concentration. Moreover, in the case of low concentration samples, leakage from formed nanoparticles can also occur. This is also in accordance with the mechanism proposed by Chow [30]. The loading capacity was instead directly related to the drug-to-polymer mass ratio, allowing to obtain the desired loading content by designing the formulation.

### 3.4. Differential Scanning Calorimetry

**Figure 4:**
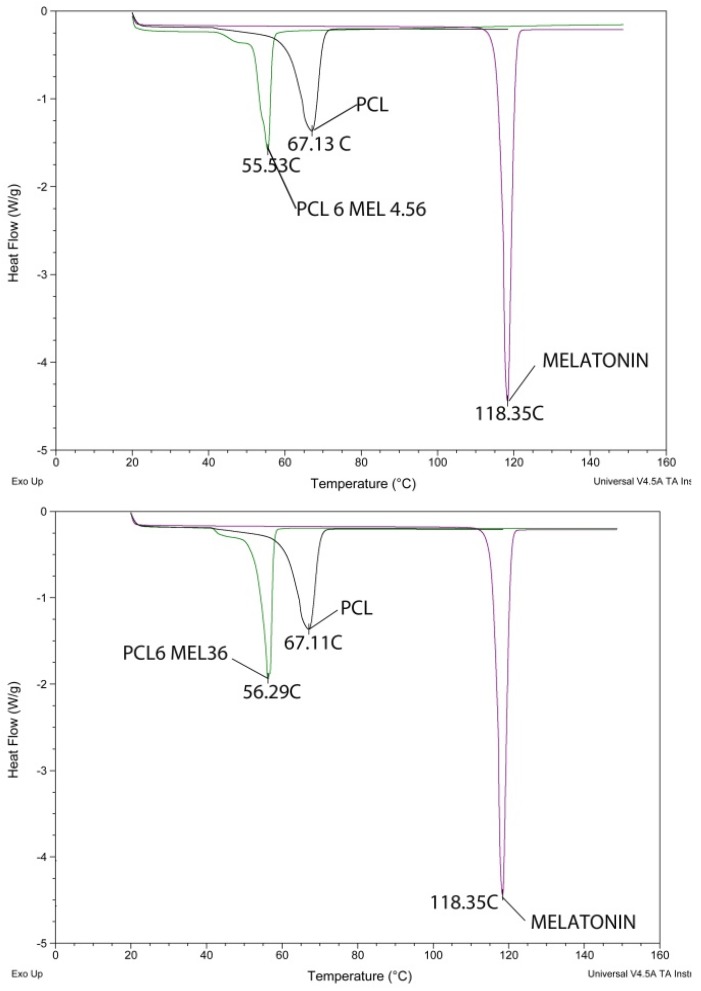
Differential scanning calorimetry (DSC) analysis results for formulation P6M4.56 (**top**) and P6M36 (**bottom**) compared with the raw materials ones.

Thermograms of pure PCL and melatonin were collected as reference. As shown in Figure 4, the individual peaks of pure PCL and melatonin were observed at 67.13 °C and 118.35 °C respectively, while in the case of MEL-loaded nanoparticles a single peak at 55 °C appears.

The absence of the active principle peak in the nanoparticle thermograms is commonly considered a proof that the drug is encapsulated in the nanoparticles and not only outside the nanoformations [53,54]. Since the DSC was performed on dried particle separated from supernatant liquid, the absence of melatonin outside the particles confirms that the increase in zeta potential discussed in Section 3.2 can be ascribed to free melatonin in the solution. Comparing the two different DSC scans of Figure 4, it is noticeable the NP peak shift from 55.53 °C to 56.29 °C for formulation PCL6MEL36; this phenomenon can be attributed to higher melatonin content in P6M36 formulation in accordance with LC and EE data.

### 3.5. Scanning Electron Microscopy

**Figure 5:**
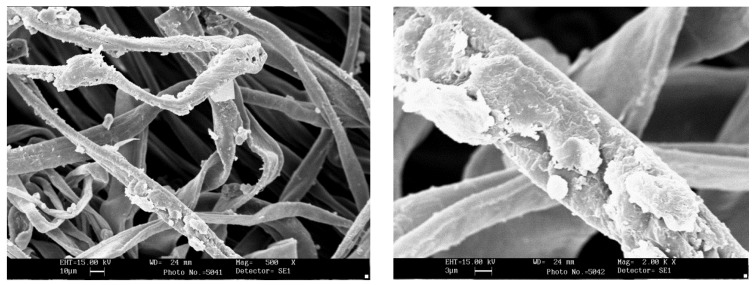
SEM image of fabric impregnated with formulation P25M4.56, at magnification 500× (**left**) and 2000× (**right**).

SEM morphological analysis was carried out on functionalized cotton fabrics. The aim was to check NP adhesion on the fibers and compare NP aggregate size with fiber size, to understand NP interaction with cotton bean shaped morphology. Samples of cotton fabrics functionalized with P25M4.56 are shown in Figure 5; the fibers are covered by polymer aggregates or by particles of micrometric size.

**Figure 6:**
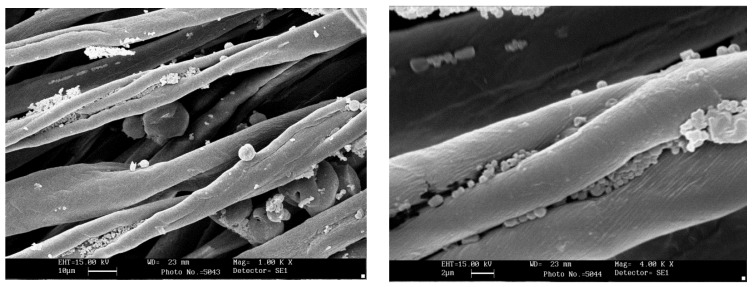
SEM image of fabric impregnated with formulation P6M36, at magnification 1000× (**left**) and 4000× (**right**).

In the case of formulation P6M36, Figure 6 shows that the smallest nanoparticles are hosted inside the dip of the typical bean shape cotton fiber, rather than being grafted on the exterior surface. Therefore, depending on the particles diameter, different extents of NPs adhesion can be achieved.

### 3.6. In Vitro Release Test

**Figure 7:**
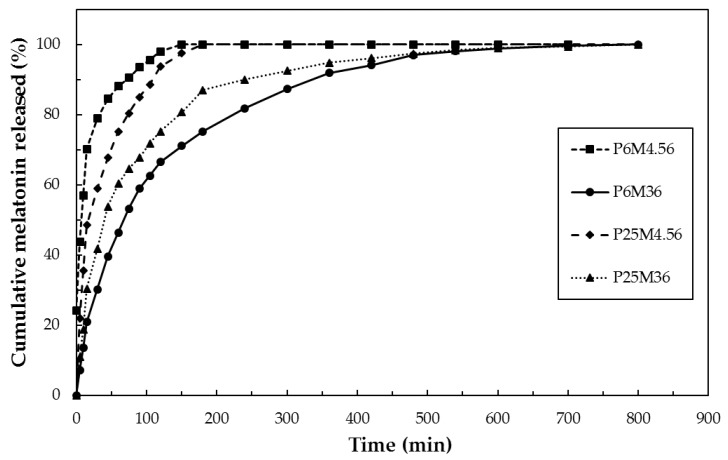
Cumulative release curves for the functionalized patches.

The cumulative release curves normalized over the total amount of melatonin released in the Franz diffusion experiment are plotted in Figure 7.

From the graph, it clearly emerges that the formulations P6M4.56 and P25M4.56, with the lower amount of melatonin, displayed a faster drug release, releasing the total amount of melatonin within 150 min. On the other hand, the systems with the higher amount of melatonin, P6M36 and P25M36, were able to release the drug for a prolonged time: in this case, the total amount of drug was released at about 500 min. This result clearly highlights that nanoparticles systems with higher drug loading can act as better drug reservoir system into the patch with a prolonged release profile.

Furthermore, the LC% and the nanoparticles sizes were found to play a key role in determining the drug release profiles. Comparing the kinetics of the two low MEL samples, it can be observed that P25M4.56 exhibited a slower kinetics, ascribable to the lower LC%, which made MEL diffusion from NP slower.

Moreover, the sizes of the system P25M4.56 were found to be 853 nm, while those of the system P6M4.56 were about 260 nm. The slower release rate from P25M4.56 could be ascribed to the higher surface area of the system P6M4.56 due to its reduced sizes. In fact, higher surface area means higher surface from which the drug can be released. 

Focusing on the comparison between the samples with higher amount of melatonin, P25M36 and P6M36, is worth of noting that the two systems presented different properties. Firstly, their sizes were very different: P25M36 was characterized by sizes in the micrometer order, about 2.3 µm, while the sizes of P6M36 were about 378 nm. It is well-established that a nanoparticles system can offer a better controlled release than that involving micro-particles. Secondly, P25M36 and P6M36 displayed different LC%: 56.4 and 84.4%, respectively. Consequently, it is reasonable that the sample P25M36 showed a faster melatonin release than P6M36, due to its micrometric sizes and low LC%.

In conclusion, the sample P6M36 was found to be the best sample, characterized by the best properties among all the prepared samples, i.e., a higher LC% value and good particles sizes. It displayed the best controlled release, acting as drug reservoir system at the skin-like surface. In fact, the particle sizes of P6M36 was in the order of the membrane cutoff. Therefore, the nanocarriers released MEL inside the membrane and then the drug was continuously released from the particles to the receptor fluid.

**Figure 8:**
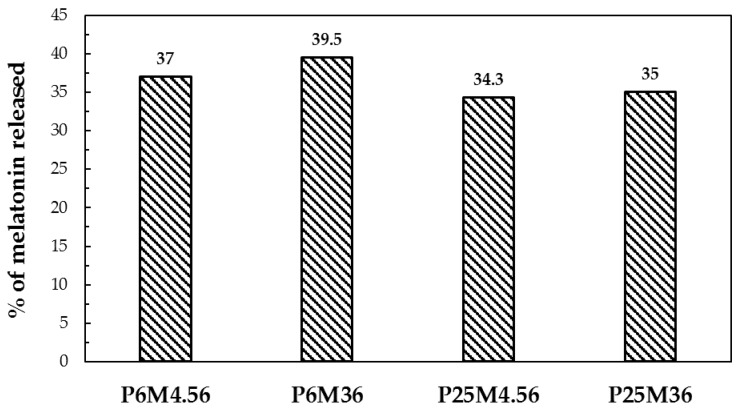
Amount of melatonin released with respect to the theoretical amount on the fabric.

The fraction of melatonin released with respect to the amount theoretically loaded on the fabric are shown in Figure 8. Less than half of the melatonin was released from the carrier: this can be explained by the fact that, as it was stated in the introduction, PCL is a polymer that biodegrades over long periods (e.g., several weeks). Thus, PCL NPs act as a drug reservoir on the fabrics.

The release curves were fitted using the semi-empirical models listed in Section 2.7; the correlation coefficients of every model for the tested formulation are listed in Table 4. 

It can be observed that the release kinetics of the functionalized systems can be fitted best by Baker–Lonsdale model, which was developed to describe diffusion controlled drug release from spherical matrices. This result gives further support to the hypothesis that melatonin was released by diffusion from the nanoparticles.

## 4. Conclusions

This work described the design and testing of a transdermal drug delivery device. Melatonin was encapsulated in PCL by mean of FNP precipitation, with effective control of particle diameter in the right size for transdermal applications. The encapsulation process was highly efficient and more than 80% of melatonin was incorporated in the polymer as proven by UV-visible and DSC analysis. Franz cell experiments demonstrated that the functionalized fabrics were an effective device for delivering melatonin through skin in a continuous and controlled way. The present research provides interesting preliminary results about the possibility of administering melatonin through daily worn garments. Such an administration approach can overcome the side effect of first pass metabolism occurring in the case of oral administration. Moreover, it represents an improvement with respect to conventional transdermal systems such as creams and ointments that must be locally applied several times a day. Once worn next to skin, the fabric can deliver melatonin for several hours without any further action of the patient to be taken.

## Figures and Tables

**Table 1:** List of the formulations produced.

Sample Name	Melatonin Concentration (mg/mL)	Polycaprolactone (PCL) Concentration (mg/mL)	Mass Ratio (MR)
P6M4.56	4.56	6	0.76
P6M12	12	6	2
P6M18	18	6	3
P6M24	24	6	4
P6M36	36	6	6
P10M4.56	4.56	10	0.456
P10M12	12	10	1.2
P10M18	18	10	1.8
P10M24	24	10	2.4
P10M36	36	10	3.6
P25M4.56	4.56	25	0.1824
P25M12	12	25	0.48
P25M18	18	25	0.72
P25M24	24	25	0.96
P25M36	36	25	1.44

**Table 2:** Model used to fit release data.

Mathematical Model	Equation
Zeroth order	*F* = k*t*
First order	Ln (1 − *F*) = −k*t*
Higuchi	*F* = k*t*1/2
Baker–Lonsdale	3/2[1 − (1 − *F*)^2/3^] − *F* = k*t*
Hixon–Crowell	1 − (1 − *F*)^1/3^ = k*t*
Square root of mass	1 − (1 − *F*)^1/2^ = k*t*
Three seconds root of mass	1 − (1 − *F*)^2/3^ = k*t*

**Table 3:** Loading capacity (LC) and encapsulation efficiency (EE) of selected formulations.

Formulation	MR	Mean Diameter (nm)	LC%	EE%
P25M4.56	0.18	852.8	13.0	81.6
P6M4.56	0.76	259.9	39.6	86.2
P25M36	1.44	2316.3	56.4	89.9
P6M36	6	378.1	84.4	90.4

**Table 4:** Coefficients of determination (R^2^) values for release data fitting.

Sample	Zeroth Order	First Order	Higuchi	Hixon–Crowell	Baker–Lonsdale	Square Root of Mass	Three Second Root of Mass
P6M4.56	0.33	0.94	0.88	0.79	0.97	0.69	0.56
P6M36	0.63	0.90	0.97	0.82	0.99	0.77	0.71
P25M4.56	−0.46	0.71	0.65	0.45	0.94	0.28	0.07
P25M36	0.84	0.95	0.98	0.92	0.99	0.91	0.88
